# Low dose of acetylsalicylic acid and oxidative stress-mediated endothelial dysfunction in diabetes: a short-term evaluation

**DOI:** 10.1007/s00592-014-0629-4

**Published:** 2014-08-06

**Authors:** Eliezer Joseph Tassone, Maria Perticone, Angela Sciacqua, Simona Fortunata Mafrici, Chiara Settino, Natalia Malara, Vincenzo Mollace, Giorgio Sesti, Francesco Perticone

**Affiliations:** 1 Department of Medical and Surgical Sciences, University Magna Græcia of Catanzaro, Campus Universitario di Germaneto, V.le Europa, 88100 Catanzaro, Italy; 2 Interregional Research Center for Food Safety and Health (IRC-FSH), Catanzaro, Italy; 3 IRCCS San Raffaele, Rome, Italy; 4 Department of Clinical and Experimental Medicine, University Magna Græcia of Catanzaro, Campus Universitario di Germaneto, V.le Europa, 88100 Catanzaro, Italy; 5 Department of Health Science, University Magna Græcia of Catanzaro, Campus Universitario di Germaneto, V.le Europa, 88100 Catanzaro, Italy

**Keywords:** Aspirin, Endothelial dysfunction, Oxidative stress, Diabetes mellitus

## Abstract

Current guidelines suggest the use of low doses of acetylsalicylic acid (ASA) for patients with diabetes mellitus (DM) in primary prevention. However, the evidences demonstrating the beneficial effect of ASA in primary prevention are conflicting. In this pilot study, we evaluated in a group of diabetic patients, in primary prevention, the impact of ASA treatment on oxidative stress and vascular function. We enrolled 22 newly diagnosed diabetic patients, without any previous clinical evidence of cardiovascular disease, to receive, in primary prevention, ASA (100 mg/daily). We tested, in basal condition, after 4 weeks of ASA administration and after 4 weeks of pharmacological washout, the impact of ASA treatment on endothelial function, assessed by a semipletysmographic method, measuring the main oxidative stress parameters related to it. As expected, after 4 weeks of treatment, ASA induced a significant reduction of plasma thromboxane-A_2_, as a consequence of cyclooxygenase-1 inhibition. By contrast, ASA significantly increased the plasma and urine 8-iso-PGF2α, a well-known prothrombotic molecule, parallel to an increase of plasma NOX2 levels. The enhancement of this oxidative pathway is associated with a significant impairment of endothelial vasodilation, assessed by reactive hyperemia index (RHI). The pharmacological washout reverted all parameters to basal condition. Our findings suggest that ASA utilization for primary prevention in diabetic patients causes a significant increase of oxidative stress burden impairing the vascular function. Present data, if confirmed on a larger population, could permanently discourage the use of the ASA for the primary prevention in patients with DM.

## Introduction

Diabetes mellitus (DM) represents one of the most important cardiovascular (CV) risk factors since diabetic patients have an increased risk, two- to fourfold greater, to develop coronary artery disease, myocardial infarction, heart failure, peripheral artery disease, and sudden death [1, 2]. Atherosclerosis is the most important pathogenetic mechanism explaining the relationship between DM and CV events. It recognizes as the *primum movens* the endothelial dysfunction, a condition characterized by the reduction of the nitric oxide (NO) bioavailability inducing the impairment of endothelium-mediated vasodilation and the activation of proinflammatory and proliferative pathways.

Hyperglycemia and insulin resistance, present in diabetic disease, induce endothelial dysfunction by several mechanisms, including the advanced glycation end products (AGEs) synthesis, the activation of polyol and hexosamine pathways, and the diabetic dyslipidemia pattern (formation of small and dense low-density lipoprotein (LDL) particles and reduction of high-density lipoprotein cholesterol (HDL-C)), all factors that promote the oxidation/inflammation processes in the vasculature wall [3, 4].

Moreover, a marked increase in oxidative stress, characterized by the overproduction of reactive oxygen species (ROS), has been observed in diabetic patients [5]. The catalytic subunit of the NADPH oxidase (NOX) enzyme NOX2 is the most important cellular source of ROS, which are able to react with various molecules. In particular, the reaction of ROS with arachidonic acid of cell membranes, leads to production of isoprostanes which, in turn, induce platelet aggregation, vasoconstriction and stimulate the inflammatory cascade in the vasculature, all pathogenic mechanisms involved in endothelial dysfunction [6, 7]. The platelet isoprostane 8-iso-PGF2α is a vasoconstrictor and platelet activator formed by non-enzymatic oxidation reaction between arachidonic acid and ROS. There are evidences demonstrating that diabetic patients have an upregulation of NOX2 compared to healthy controls, thus presenting higher levels of ROS and 8-iso-PGF2α [8].

Acetylsalicylic acid (ASA), an inhibitor of cyclooxygenase-1 (COX1) that prevents platelet thromboxane (Tx)-A_2_ formation [9, 10], used at low dose (100 mg/daily) in secondary CV prevention, is able to reduce clinical events in patients with coronary and peripheral artery disease and DM. In fact, platelet Tx-A_2_ overproduction, and the subsequent reduction obtained with aspirin administration, has been demonstrated in diabetic patients [11]. Because diabetic patients are characterized by a high CV risk profile, similar to those who have already had a major CV event (myocardial infarction or stroke), it has been proposed the use of ASA for patients with DM also in primary prevention [12, 13]. The American Diabetes Association, for example, recommends the use of ASA in primary prevention in diabetic patients with 10-year CV risk >10 % (men >50 years or women aged >60 presenting at least one additional major risk factor, such as family history of coronary artery disease, smoking, dyslipidemia, hypertension, or albuminuria) [14]. However, the beneficial effect of ASA in primary prevention is still unclear [15]. In fact, there are recent evidences that attribute to ASA a negative effect on oxidative stress-related parameters in diabetic patients [16]. Particularly, an oxidative stress-mediated platelet isoprostane overproduction occurs in aspirin-treated diabetic patients that is associated with the attenuation of aspirin-mediated Tx-A_2_ inhibition, effect promoting platelet recruitment. Moreover, the NOX2 upregulation observed in diabetic subjects contributes to the ASA-induced formation of 8-iso-PGF2α in both platelets and urine [8] that, in turn, increases the burden of oxidative stress.

Although increasing evidences demonstrate a negative impact of ASA on oxidative stress, in primary prevention in diabetic patients, it remains uncertain whether this biological effect is followed, in turn, by functional alterations that contribute to an increased risk of developing atherosclerosis. Because these contrasting evidences, in a recent statement, the FDA expressed an unfavorable opinion to the use of ASA for the primary prevention of heart attack and stroke (http://www.fda.gov/Drugs/ResourcesForYou/Consumers/ucm390574.htm), by underlying the serious risks of bleeding in the stomach and brain associated with the use of ASA without an effective therapeutic usefulness. Thus, in this study, we evaluated, in a group of newly diagnosed type 2 diabetic patients, the effect of ASA on endothelium-dependent vasodilation and oxidative stress burden, testing the hypothesis that ASA administration (100 mg/daily) in primary prevention is able to increase the oxidation parameters leading to endothelial dysfunction.

## Materials and methods

### Study population

According to statistical sample size calculation, we enrolled 22 newly diagnosed type 2 diabetic patients, diagnosed by an oral glucose tolerance test (OGTT) [17], without any clinical evidence of CV diseases. We excluded patients with history of acute vascular events, cardiac arrhythmia or congestive heart failure, type 1 DM, chronic kidney disease or serum creatinine level ≥1.5 mg/dL, history of cancer, infections, smoke, chronic treatment with non-steroidal antiinflammatory drugs, statins, and antiplatelet drugs, in the previous 6 months. At the time of enrollment and during the whole study, all patients were free from any pharmacological treatment. After the enrollment, patients started a 4-week treatment with 100 mg/daily of ASA (T1), followed by a washout period of 4 weeks (T2). The assessment of endothelial function and oxidative stress parameters were performed at baseline and at the end of T1 and T2. Adherence to the treatment was assessed by the pill-count method. The Ethical Committee of University *Magna Graecia* of Catanzaro approved the protocol, and informed written consent was obtained from all participants. All the investigations were performed in accordance with the principles of the Declaration of Helsinki.

For this study, we did not include the control group for two reasons: at first, because patients were control of themselves; secondly, we preferred not exposing subjects without indications for ASA treatment to a possible bleeding risk.

### Laboratory analyses

#### Materials

All materials were from Sigma-Aldrich unless otherwise specified.

#### Blood sampling

After a 12-h fast, blood samples were taken. At 08:00 a.m., patients underwent routine biochemical analysis including total cholesterol, HDL cholesterol, LDL cholesterol, triglyceride, glucose, and creatinine. Samples, obtained from patients after supine rest for at least 10 min, were taken in tubes with 3.8 % sodium citrate and centrifuged at 300 rpm for 15 min to obtain supernatant. Plasma samples were immediately stored at −80 °C.

#### Tx-A_2_ and NOX2 quantification

Human Tx-A_2_ Elisa Kit (Bio-medical assay) and enzyme-linked immunosorbent assay kit for cytocrome b-245 β polypeptide (cloud-clone corp) were used, respectively, for plasma Tx-A_2_ and NOX2 quantitative determinations. Serial dilutions at different concentrations of the standard are prepared. Samples are centrifuged at 1,000 rpm for 10 min, and supernatants are used for the assay. The human Tx-A_2_ and NOX2 polyclonal antibodies are pre-coated into 96-well plate. Hundred microliters of standard or samples was added into each well and incubated 90 min at 37 °C. Plate was washed twice with TBS washing buffer; then, 100 µl of working solution, containing biotinylated antihuman Tx-A_2_ and antihuman NOX2 detection antibodies (dilution 1:100), was added and incubated at 37 °C for 60 min. The plate was washed with TBS 3 times and, after 100 µl Avidin–Biotin–Peroxidase Complex (dilution 1:100) addiction, incubated at 37 °C for 30 min. The plate was washed 5 times with TBS, and 90 µl of TMB color developing agent was added, and then the plate was incubated at 37 °C for 15 min, in the absence of light. At the end, 100 µl of stop solution is added to stop the reaction. The intensity of the color change was measured spectrophotometrically with Victor™ X4 multi-label plate reader at the wave length of 450 nm. The standard curve was plotted as the relative optical density (OD) 450 of each standard solution versus the respective concentration of the standard solution. The human Tx-A_2_ and NOX2 concentration of the samples were interpolated from the standard curve, using curve expert 1.4.

#### Human 8-isoprostane quantification

Oxiselect™ 8-iso-prostaglandin F2α (8-iso-PGF2α) ELISA kit (Cell Biolabs, inc.) was used for quantitative determination of human 8-isoprostane in plasma and urines. Hydrolysis of lipoprotein or phospholipid coupled 8-iso-PGF2α is required to measure both free and esterified isoprostane. To hydrolyze these ester bounds, plasma was treated with 1 part of 10 N NaOH for every 4 parts of liquid samples and incubated for 2 h at 45 °C. Then, 100 µl of 10 N HCl per 500 µl of hydrolyzed sample is added. These samples were centrifuged for 5 min at 12,000 rpm, and supernatant was used in the assay. Urine samples were acidified to pH 3 by adding 1/10 volume of 1 N HCl. Different serial dilutions of the standard were prepared. Hundred microliters of diluted antiisoprostane antibody (1:1,000) was added to the goat antirabbit antibody coated plate and incubated 1 h at 25 °C on an orbital shaker. The plate was washed 5 times with wash buffer. Fifty-five microliters of standard or samples and 55 µl of 8-isoprostane-HRP conjugate were combined in a microtube, mixing thoroughly. Hundred microliters of the combined solution was transferred per well and incubated 1 h at 25 °C on an orbital shaker. The plate was washed 5 times with wash buffer; then 100 µl of substrate solution was added to each well and incubated at room temperature for 20 min on an orbital shaker. The enzyme reaction was stopped by adding 100 µl of stop solution to each well. Immediately, we read absorbance of each well on Victor™ X4 multi-label plate reader at the wave length of 450 nm. The standard curve was plotted as the relative (OD) 450 mm of each standard solution versus the respective concentration of the standard solution. The human 8-isoprostane concentration of the samples was interpolated from the standard curve.

### Endothelial function

Endothelial function was evaluated by a semipletysmographic method, with the measurement of digital pulse volume amplitude (PVA). PVA was measured with the subjects placed in the supine position, in a quiet, temperature-controlled environment set at 22 °C. A peripheral arterial tonometer (PAT) was used to measure PVA in the fingertip of the index finger (Itamar-Medical, Caesarea, Israel). The peripheral arterial tonometer apparatus consists of a finger-mounted probe that surrounds the fingertip with an electronically controlled, inflatable, pressurized air cushion confined within a rigid external case. The pressure changes within the probe that accompany PVA changes in the fingertip is transmitted to a personal computer where the signal is band-pass filtered (0.3–30 Hz), amplified, displayed, and stored. PVA was analyzed at rest and during reactive hyperemia (RH). RH was elicited by the release of an upper arm blood pressure cuff inflated above systolic pressure for 5 min. Digital PVA–RH was calculated as the ratio of the average PVA over a one-minute time interval starting 1 min after cuff deflation (RH) divided by the average PVA measured for 1 min before cuff inflation (baseline) (RH index, RHI). The PVA from the index finger of the other, non-ischemic hand (which was not subject to RH), was measured continuously throughout the study to assess any drift in the magnitude of the signal due to systemic factors.

### Statistical analysis

We computed the minimum sample size with respect to a two-tailed one-sample Student’s *t* test, considering: a difference for RHI variation to be detected between baseline and after ASA treatment, |d| ≥15 %; standard deviation of the paired differences, SD = 0,11; and type 1 error probability *α* = 0.05 and power 1_b = 0.90 (0.80). Differences among different times for the oxidative stress parameters, (in particular, urinary and plasma 8-iso-PGF2α, plasma Tx-A_2_ and NOX2), and vascular function were evaluated by ANOVA for repeated measures. Chi-squared test will be utilized for categorical variables. Data are reported as mean ± SD. Differences were assumed to be significant at *P* < 0.05. All comparisons were performed using the Statistical Package of Social Science (SPSS) version 16.0 for Windows (SPSS Inc., Chicago, IL, USA).

## Results

### Study population

In Table 1, we reported the anthropometric, biochemical, and hemodynamic characteristics of the study population, expressed as mean ± SD. As evident, the group consists of 11 men and 11 women (mean age 59.6 ± 6.5 years) with mean blood pressure values of 134.7/83.4 mmHg. Moreover, lipid profile showed mild hypercholesterolemia and hypertriglyceridemia, consistent with their clinical condition of DM. No significant differences were observed at the end of study (Table 1).

**Table 1:** Anthropometric, biochemical, and hemodynamic characteristics of the study population in basal

	Basal	End of study	*P*
Gender (m/f)	11/11	11/11	–
Age (years)	59.6 ± 6.5	59.6 ± 6.5	–
BMI (kg/m^2^)	29.3 ± 2.6	29.0 ± 3.1	0.808
SBP (mmHg)	134.7 ± 11.0	130.5 ± 14.7	0.457
DBP (mmHg)	83.4 ± 9.0	81.0 ± 11.4	0.590
PP (mmHg)	51.3 ± 9.7	49.5 ± 12.8	0.714
Total cholesterol (mg/dl)	208.8 ± 22.5	202.3 ± 17.7	0.460
HDL cholesterol (mg/dl)	42.7 ± 10.7	38.7 ± 10.3	0.382
LDL cholesterol (mg/dl)	139.2 ± 23.7	141.8 ± 26.5	0.811
Triglyceride (mg/dl)	134.5 ± 36.8	108.5 ± 42.7	0.142
Fasting glucose (mg/dl)	124.0 ± 38.0	122.4 ± 24.2	0.907
Fasting insulin (mU/ml)	20.9 ± 6.6	19.3 ± 4.9	0.526
HOMA index	6.0 ± 1.7	5.9 ± 1.4	0.882
HbA1c (%; mmol/mol)	6.5 ± 0.9; 48.0 ± 2.7	6.4 ± 1.2; 46.0 ± 3.6	0.827
Creatinine (mg/dl)	0.9 ± 0.4	0.8 ± 0.7	0.685

*BMI* body mass index, *SBP* systolic blood pressure, *DBP* diastolic blood pressure, *PP* pulse pressure

### Effects of ASA treatment on Tx-A_2_ and 8-iso-PGF2α levels

**Fig. 1:**
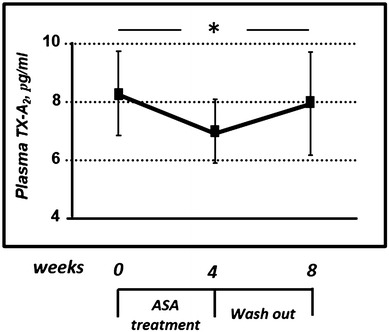
Effects of ASA on plasma levels of Tx-A_2_ in diabetic patients. ASA treatment induced a significant reduction of Tx-A_2_ levels, as consequence of COX1 inhibition. * = *P* < 0.05 by ANOVA for repeated measures

**Fig. 2:**
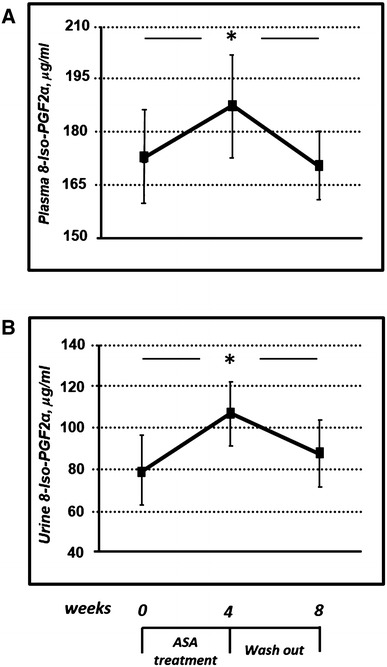
Effects of ASA on urinary **a** and plasma **b** levels of 8-iso-PGF2α in diabetic patients. ASA treatment was able to induce a significant increase of 8-iso-PGF2α levels with a reduction after 4 weeks of drug washout. * = *P* < 0.05 by ANOVA for repeated measures

The antiplatelet effect of ASA, evaluated by plasma Tx-A_2_ measurement, was observed after 4 weeks of treatment (from 8.25 ± 1.45 to 6.95 ± 1.08 pg/ml, *P* = 0.013). At the end of pharmacological washout, Tx-A_2_ levels significantly increased, reaching almost basal values (Fig. 1). As observed in Fig. 2, treatment with ASA 100 mg/day induced a significant increase in plasma (from 173.0 ± 13.5 to 187.5 ± 14.8 μg/ml, *P* = 0.009) and urine (79.5 ± 17.5 to 107.0 ± 15.5 μg/ml, *P* < 0.0001) 8-iso-PGF2α, respectively. Discontinuation of treatment for 4 weeks (washout) resulted in a significant reduction in 8-iso-PGF2α levels, similar to that observed in baseline conditions (Fig. 2).

### Effects of ASA treatment on NOX2 levels

**Fig. 3:**
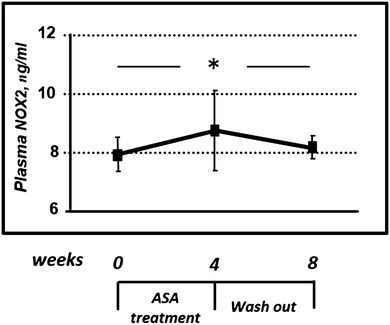
Effects of ASA on NOX2 plasma levels in diabetic patients. ASA treatment was able to induce a significant increase of NOX2 levels, which returned to basal values after 4 weeks of pharmacological washout. * = *P* < 0.05 by ANOVA for repeated measures

Four weeks administration of ASA was able to induce a NOX2 overexpression. In particular, NOX2 levels increased in plasma samples, from 7.93 ± 0.57 to 8.75 ± 1.38 ng/ml (*P* = 0.010). Interestingly, after the washout period, NOX2 levels decreased, returning nearly to basal levels (Fig. 3).

### Effects of ASA treatment on endothelial function

**Fig. 4:**
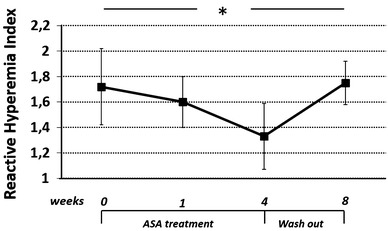
Effects of ASA on endothelium-dependent vasodilation assessed by reactive hyperemic index (RHI). ASA treatment in diabetic subjects caused a significant reduction of endothelial vasodilatory response to ischemia; after the washout period, RHI values were similar to those observed in basal condition. *P* < 0.0001 by ANOVA for repeated measures

Effects of ASA treatment on vascular function, expressed by RHI, are shown in Fig. 4. As observed, the administration of 100 mg/day of ASA induced, already after 1 week, a significant reduction of the vasodilatory response to ischemia that reached the maximal reduction after 4 weeks of treatment (RHI from 1.72 ± 0.30 to 1.33 ± 0.26, *P* < 0.0001); normal value ≥ 1.67. After 4 weeks of pharmacological washout, endothelial function was completely restored, with average values of RHI similar to that observed in basal conditions (RHI = 1.75 ± 0.17).

## Discussion

**Fig. 5:**
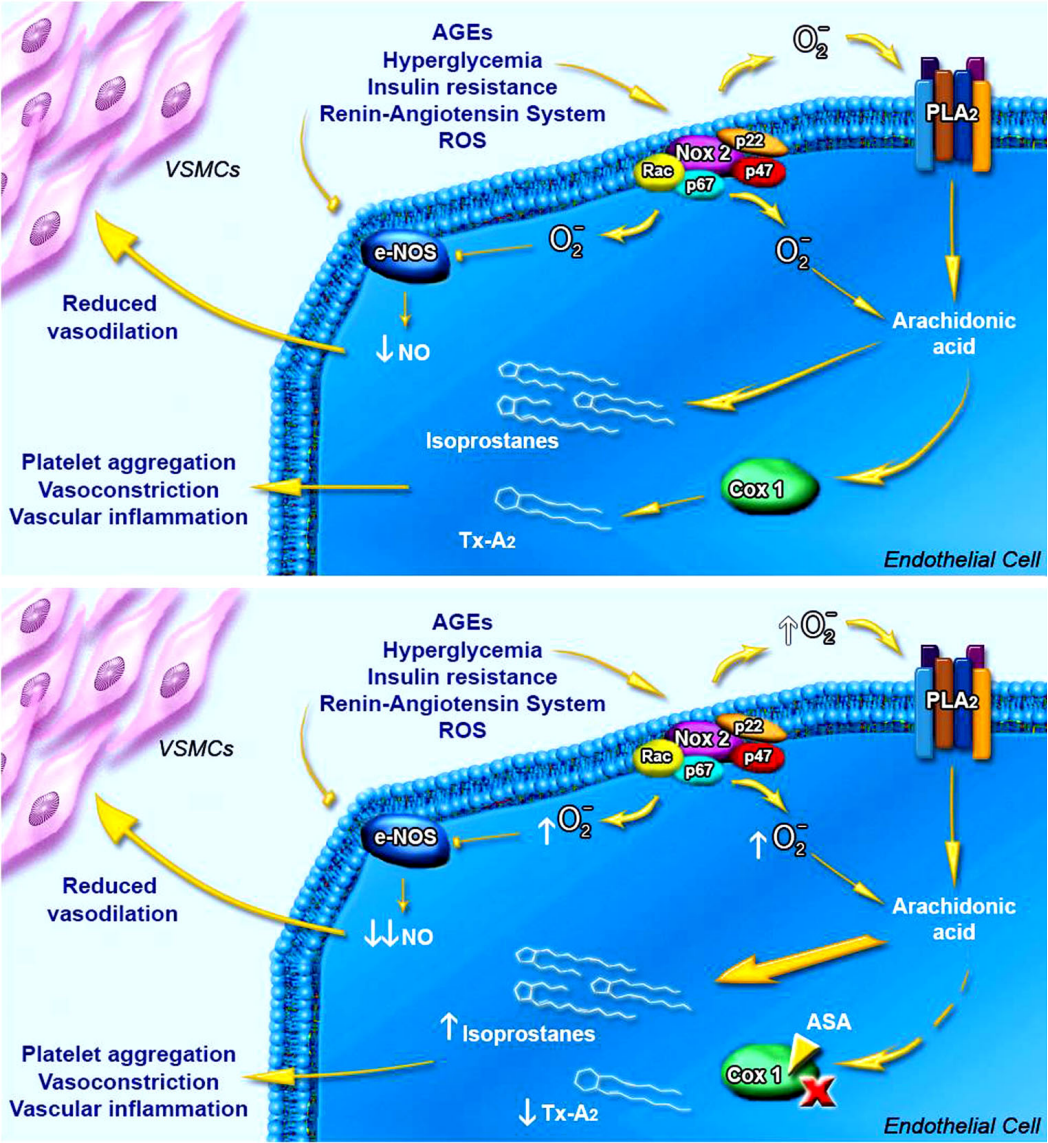
Diagram graphically reports the effects of ASA on vascular oxidative stress leading to endothelial dysfunction. COX1 inhibition triggers an escape pathway that is able to promote the production of isoprostanes that, in turn, contribute to impair the endothelium-dependent vasodilation

In this study, we evaluated, in a group of newly diagnosed type 2 diabetic patients, the impact of ASA treatment in primary prevention on oxidative stress and vascular function. We demonstrated that ASA administration induces a significant increase in global oxidative burden, as shown by the increase of plasma and urine 8-iso-PGF2α, a well-known proaggregating and prothrombotic molecule [18]. As already speculated, the increase of 8-iso-PGF2α could be promoted, indirectly, by the ASA-induced inhibition of the COX1 enzyme that, in turn, leads to the triggering of an escape pathway of isoprostanes formation [16, 19]. Moreover, in diabetic patients, there is *per se* an increased activity of the NADPH oxidase that is responsible for the increased production of ROS [8], which are able to interact with arachidonic acid to form isoprostanes [20]. ASA treatment is also able to increase NOX2 levels, contributing to cellular oxidative burden in ROS production. In this way, the treatment with ASA, by selectively inhibiting COX1, prevents the arachidonic acid contribution to generate Tx-A_2_, favoring the switch of the substrate for the isoprostanes production [21]. According to this, our data show that ASA induces a significant reduction in plasma Tx-A_2_ and increases the plasma and urinary levels of 8-iso-PGF2α (Fig. 5). Of interest, Gonçalves and corkers recently reported that concomitant use of metformin and ASA in diabetic patients can reduce levels of urinary biomarkers of oxidative stress [22], offering new important therapeutic implications for antidiabetic drugs.

The most relevant data of our study are the demonstration that ASA treatment in type 2 diabetic patients causes a significant reduction in the endothelium-dependent vasodilatory response. The reduction of RHI, already occurred after 1 week of treatment, which reached pathological values after 4 weeks, demonstrates that the ASA-increased oxidative stress could adversely affect the vascular function, by inhibiting its vasodilatory properties in a setting of patients considered at high CV risk also in primary prevention. This finding has an important clinical implication, because endothelial dysfunction represents a powerful negative prognostic factor in different setting of patients, including the uncomplicated hypertensive patients that are considered at low risk in comparison with diabetic ones [23]. Moreover, the impairment of endothelial function might worse the metabolic control since endothelial dysfunction interacts with some different molecular pathways involved in the appearance of new diabetes [3, 24].

These results are clinically relevant because, according to current evidences of the literature, there is still no unanimous opinion about the real need of ASA treatment for the primary cardiovascular prevention [25]. Our data show that in DM, in which oxidative stress plays a pivotal role in the pathogenesis of vascular damage, the treatment with aspirin may increase the oxidative stress burden, worsening in turn the vascular function. This negative effect of ASA could have an important impact, especially in diabetic patients who undergo percutaneous coronary angioplasty and stenting, for whom the association of ASA with another antiplatelet agent is recommended. This treatment, on the basis of our findings, could be potentially dangerous because it reduces the NO bioavailability with a consequent worsening of vascular function. Moreover, our results contribute to clarify an important pathophysiological mechanism with a relevant clinical application because they consent to better interpret the controversial results obtained in primary prevention of diabetic patients [15]. This is further supported by the fact that ASA, beyond the observed negative impact on vascular function, increases the bleeding risk, mainly in subjects without previous CV events in which a clear benefit of ASA utilization is not clearly demonstrated. Our results differ from those reported by Raghavan et al. [26], but these differences are due to the methods and study population characteristics. In fact, these Authors evaluated diabetic patients treated with statins, antidiabetic, and antihypertensive drugs, while our patients were newly diagnosed and without any pharmacological treatment. In addition, the Authors evaluated total antioxidant status and blood total glutathione, while we investigated the specific pathway involving NOX2 that is overexpressed in diabetic patients.

Thus, present data, if confirmed on a larger study population, could permanently discourage the use of the ASA for the primary prevention in patients with DM, providing also new targets for future research in the prevention and treatment of oxidative stress and endothelial dysfunction related to it.

### Study limitations

The most relevant limitation of the study is the small number of patients, even if the statistical power size calculation is satisfied. This is due to the fact that the patients were enrolled at the first time of diagnosis of DM, after performing an OGTT. In addition, the aim of this study was to investigate a pathophysiological mechanism; thus, this study could be considered a pivotal one, and not an interventional one. The short duration of observation period (4 weeks) could be another limitation of the study. However, the demonstration that ASA induces a short-term negative effect, by the increase of oxidative stress and related endothelial dysfunction, could have relevant pathophysiological implications in the initial phases of vascular injury, such as in the restenosis after percutaneous coronary intervention.
